# Optimizing utilization of point source and atmospheric carbon dioxide as a feedstock in electrochemical CO_2_ reduction

**DOI:** 10.1016/j.isci.2022.104270

**Published:** 2022-04-18

**Authors:** Alex Badgett, Alison Feise, Andrew Star

**Affiliations:** 1 Strategic Energy Analysis Center, National Renewable Energy Laboratory, 15013 Denver West Parkway, Golden, CO 80401, USA; 2 Department of Chemistry, University of Toronto, Toronto, ON M5S 3J6, Canada; 3 Chemistry and Nanoscience, National Renewable Energy Laboratory, 15013 Denver West Parkway, Golden, CO 80401, USA

**Keywords:** Electrochemical energy production, Energy resources, Energy sustainability

## Abstract

The electrochemical reduction of carbon dioxide is a potential pathway for production of fuels and chemicals that uses atmospheric carbon dioxide as a feedstock. Here, we present an analysis of the potential for carbon dioxide from point sources and via direct air capture to be utilized in electrochemical reduction under different market scenarios. We show that developing a network for production of these products at scale requires capture and utilization of significant portions of the carbon dioxide that is currently emitted from large stationary point sources. Because carbon dioxide point sources are spatially and compositionally variable, their use for carbon dioxide reduction depends on electricity prices, capture cost, and location. If the power sector in the United States is decarbonized, carbon dioxide supply decreases significantly, increasing the importance of utilizing other carbon dioxide streams, and increasing the likelihood that direct air capture plays a role in supplying carbon dioxide feedstocks.

## Highlights

• Economic optimization of carbon dioxide (CO_2_) reduction in the U.S. is modeled
• Point source CO_2_ could be used to create fuels and chemicals in various regions
• As industrial sectors decarbonize, supply of CO_2_ point sources diminishes
• Direct air capture of CO_2_ could provide supplemental feedstock for product synthesis

## Graphical abstract

**Figure:**
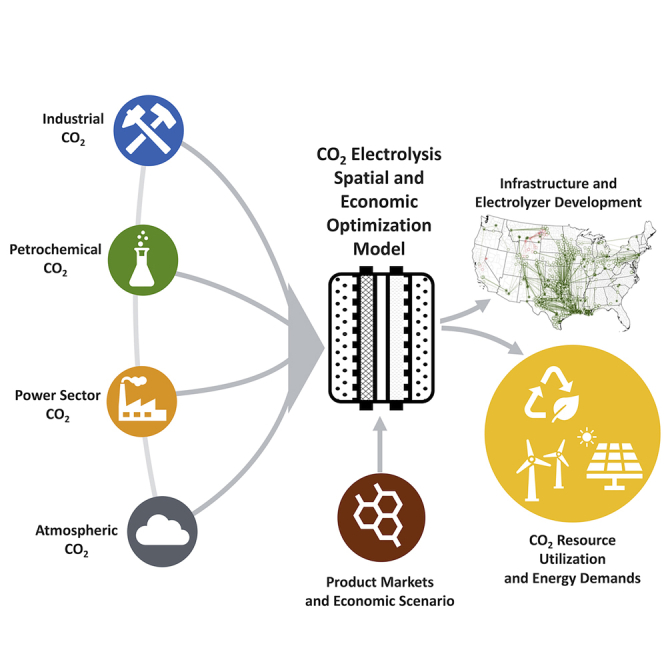

## Introduction

Of the 6.67 billion metric tons (t) of CO_2_ equivalents emitted annually in the United States, over 1.4 billion t equivalent (21%) is from the industrial sector (U.S. EPA, 2020). Decarbonizing industry is difficult, as many industrial processes require natural gas feedstocks, high temperatures, and/or high-grade heat (Davis et al., 2018). In many cases, natural gas is used as a fuel to generate heat and/or power and as a chemical reactant for production of higher value chemicals (U.S. EIA, 2014). In the United States, the organic chemical sector (e.g., plastics and rubber products) alone consumes 6.96 EJ of energy annually, which represents roughly 6.5% of the total energy consumption in the entire country (U.S. EIA, 2018). Renewable energy technologies such as wind and solar photovoltaics cannot be directly substituted into these processes, and systems are needed that transform electrons from renewable energy into molecules useful in industrial processes (Henry et al., 2020).

One opportunity is converting waste CO_2_ into chemicals and fuels via electrochemical carbon dioxide reduction (CO_2_R) (De Luna et al., 2019). CO_2_R consumes electricity, water, and CO_2_ as feedstocks into the reactor, and can produce various carbon-based molecules. Depending on the catalyst and system architecture, state-of-the-art systems can produce molecules with one to three carbons (C_1_–C_3_) at varying selectivities, current densities, and efficiencies (Bushuyev et al., 2018). Researchers have identified C_1_ and C_2_ molecules as key products of interest, as they realize high market prices and can generally be produced at higher efficiencies than more complex molecules in current systems (Bushuyev et al., 2018; De Luna et al., 2019). New and more efficient catalysts (Burdyny and Smith, 2019), membranes (Kaczur et al., 2018), and system architectures have been developed in recent years (Weekes et al., 2018), further driving the performance of CO_2_R to compete with conventional synthesis methods currently used in industry. The need for scale-up of these systems to industrially relevant levels has also been emphasized (Smith et al., 2019) as existing processes currently operate at significantly larger scales.

Recent technoeconomic analyses have considered the current performance of CO_2_R and market prices for various molecules to understand what the economic drivers of this process might be at scale (Bushuyev et al., 2018; Jouny et al., 2018; De Luna et al., 2019; Grim et al., 2019). CO_2_R requires significant amounts of electricity to reduce CO_2_ into the desired product, thus the cost of electricity is a key driver of production costs. Reducing these production costs usually involves operation of the electrolyzer at high current density and faradaic efficiency to reduce capital costs and increase efficiency. In addition, focusing on synthesizing products that can be sold at high market prices can increase the probability of cost-competitive production (Jouny et al., 2018). Recent work has also considered the production of fuels and chemicals from a CO_2_ point source perspective, optimizing the system to utilize highest impact CO_2_ streams (von der Assen et al., 2016) and mapped the total production potential from fossil and biogenic CO_2_ streams (Hansson et al., 2017). Previous analyses have also discussed the relationships between CO_2_ point sources and CO_2_R product markets, finding that market sizes and prices are key drivers of the economic feasibility of CO_2_R products (De Luna et al., 2019). Recent work has also characterized the close connections between CO_2_R technologies and existing chemical processes and infrastructure. Since many high-concentration CO_2_ streams are produced from chemical processes and CO_2_R products include multiple organic chemicals, near-term process integration opportunities exist in this space (Barecka et al., 2021a, 2021b).

Finally, life cycle analyses have been conducted on CO_2_R processes, finding that when powered with renewable electricity from wind or solar, these processes have the opportunity to reduce greenhouse gas emissions significantly relative to conventional synthesis pathways (Rosental et al., 2020; Sadok et al., 2020). The overall environmental impact of CO_2_R products will vary greatly based on the type of source stream CO_2_, the carbon intensity of the energy supply, and the possibility for use of low-carbon transportation and chemical manufacturing facilities. A system-level life cycle analysis of CO_2_R product impacts is outside the scope of this work but is an area for future work to investigate.

A concentrated feedstock stream is required for sustained operation of a CO_2_R system. Research efforts are underway to develop systems capable of utilizing low concentration CO_2_ streams; however, current systems use concentrated feedstocks. In this work, we refer to high-concentration CO_2_ streams as those with concentrations greater than 90%. Recent progress on CO_2_R systems utilizing low concentration CO_2_ streams in addition to those that are tolerant to specific impurities has demonstrated potential; however, due to the lower maturity of these technologies, they are not considered in this analysis (Kim et al., 2015; Kumagai et al., 2019; Williams et al., 2019; Xu et al., 2020). Although the United States emits over 4 billion t of CO_2_ annually (U.S. EPA, 2020), only about 2.71 billion t of this CO_2_ is emitted from large point sources (U.S. EPA, 2019) and could be captured and purified for use as CO_2_R feedstock. Of these large point sources, only ethanol plants, ammonia plants, and natural gas processing facilities emit highly concentrated CO_2_ streams. The power sector, which comprises most other point source emissions, usually emits CO_2_ at concentrations on the order of 10% (IPCC, 2005). If CO_2_R is to be deployed at scale, an understanding of the feasibility of capturing, purifying, and allocating CO_2_ from existing point sources to CO_2_R reactors is needed.

Here we present a spatial and economic optimization modeling framework that is used to analyze CO_2_R processes and feedstock supply streams. This model allocates CO_2_ streams from point sources to sink locations where they can be used in CO_2_R systems, optimizing the system to utilize CO_2_ at the lowest possible cost. This work considers ethylene, formate, and carbon monoxide (C_2_H_4_, HCOO^−^, and CO, respectively) as possible products from CO_2_R in the United States. These products are currently consumed in significant quantities, and they can be produced from a single-step CO_2_R system with high faradaic efficiency and current density. Using this framework, we build an understanding of how CO_2_R could be optimally deployed and integrated into existing and future infrastructure at locations across the United States, excluding Alaska and Hawaii. We use national data of CO_2_ sources and organic chemical production along with the optimization model to understand where and how CO_2_R might be developed. An analysis of the spatial challenges related to CO_2_R infrastructure is presented, including the proximity of CO_2_R to existing industrial systems and their potential health and social impacts. Developing a spatially distributed network of chemical production via CO_2_R could increase resilience by decentralizing chemical production and transportation networks while maximizing utilization of CO_2_ that would be otherwise emitted to the atmosphere. The optimization platform is used to model both the role direct air capture (DAC) could play in providing supplemental CO_2_ streams and the impacts of advances in DAC technology. Finally, electricity demands associated with development of CO_2_R at scale are considered.

## Results

### CO_2_ allocations

**Figure 1:**
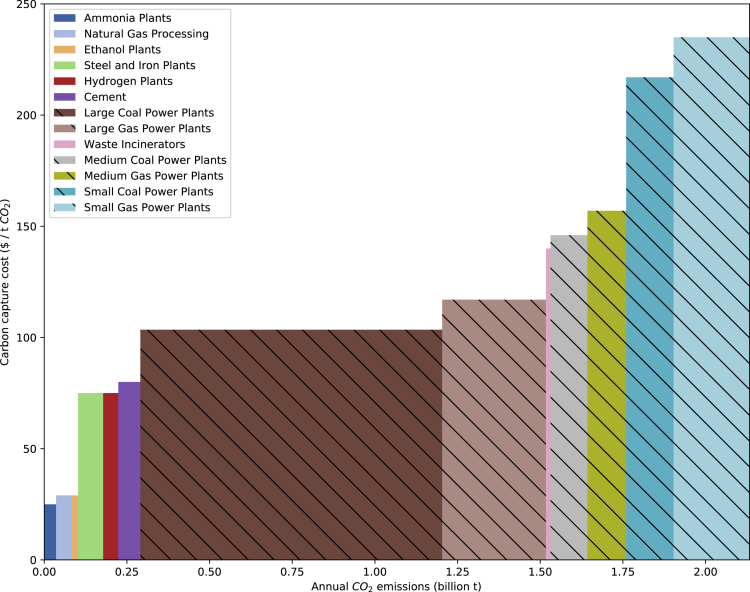
National (United States) supply curve for CO_2_ feedstocks based on estimated cost of CO_2_ capture specific to the type of point source Sections of supply curve with crosshatching indicate fossil fuel point sources from the electric power sector.

Point sources of CO_2_ and the estimated cost per metric ton ($/t CO_2_) for capturing and purifying these streams vary by the composition and source of the stream. A supply curve for CO_2_ capture and purification shown in Figure 1 illustrates how costs vary by source of CO_2_, using CO_2_ point source data from the United States Environmental Protection Agency and carbon capture costs from the National Petroleum Council (National Petroleum Council, 2019; U.S. EPA, 2019). Costs of capturing CO_2_ are adopted from the National Petroleum Council, and they generally decrease as CO_2_ concentrations in exhaust gases increase (Table S2) (National Petroleum Council, 2019). Although the total supply of point source CO_2_ is likely available to meet demand for all CO_2_R products including losses from faradaic efficiencies less than 100%, it is not necessarily available at costs and locations that facilitate its use in CO_2_R.

The CO_2_ sources included here could provide ∼24 times the CO_2_ needed to produce all the C_2_H_4_, HCOO^−^, and CO currently produced in the United States via CO_2_R, albeit this is without consideration of site-specific sizing and spatial constraints. Electric power sector sources represent about 85% of the total CO_2_ point source emissions inventoried, further emphasizing the importance of optimizing the allocation of available CO_2_. Ammonia, natural gas processing, and ethanol plants emit significantly less CO_2_; however, these streams have higher CO_2_ concentrations and are therefore less expensive to capture and purify, making them favorable feedstocks for CO_2_R. These point sources have conventionally been considered near-term candidates for supplying CO_2_ to CO_2_R. While these high concentration CO_2_ streams are the lowest cost feedstocks for CO_2_R, they are available in significantly lower quantities than other streams. The scope of these processes is more complex than the simplistic representation in this work, with each plant leveraging highly integrated and interdependent processes (Bains et al., 2017). In the optimization model, high concentration CO_2_ streams are generally utilized first due to their lower carbon capture cost, with lower concentration streams utilized in whatever extent is necessary to meet remaining product supply.

Several CO_2_R market scenarios are considered in this work, each with a unique set of assumptions and model constraints (Table 1) which affect the number, size, and spatial distribution of CO_2_R systems. In all scenarios except for the high-concentration feedstock scenario, the model is required to produce each CO_2_R product at rates equal to existing supply chains. In the high concentration scenario, the CO_2_ available is less than the amount needed for equal current production rates, and the model is constrained to utilize all of the available CO_2_.

**Table 1:** Summary of model constraints and supply chain design for scenarios modeled

Scenario name	CO_2_R location	Product supply location	Feedstock source constraint	CO_2_R size constraint
Base	At CO_2_ source	Existing manufacturing facilities	No DAC	500 MW
Distributed	At supply location	Existing petroleum terminals	No DAC	500 MW
Distributed decarbonized	At supply location	Existing petroleum terminals	No coal or natural gas power plants, no DAC	500 MW
Distributed decarbonized DAC $300/t	At supply location	Existing petroleum terminals	No coal or natural gas power plants	500 MW
Distributed decarbonized DAC $100/t	At supply location	Existing petroleum terminals	No coal or natural gas power plants	500 MW
Distributed decarbonized DAC $50/t	At supply location	Existing petroleum terminals	No coal or natural gas power plants	500 MW
High-concentration feedstock	At supply location	Existing petroleum terminals	Only ammonia, ethanol, and natural gas processing plants	No constraint^a^

a A constraint on the size of CO_2_R system was not included for the high-concentration feedstock scenario to enable the optimization model to find a feasible solution subject to other feedstock and distance constraints.

Current market sizes for product molecules are based on data gathered from publicly available sources. Market data indicate that C_2_H_4_ has the largest U.S. market size of 27 billion kg supplied per year, compared to 0.056 and 1.76 billion kg per year for HCOO^−^ and CO, respectively (Table S1) (United States International Trade Commission, 2020). The maximum CO_2_R system size is constrained to 500 MW in these scenarios, which serves as a constraint on the amount of CO_2_ that can realistically be utilized at a single location.

Development of CO_2_R in the near-term could depend on the ability to leverage existing infrastructure for storage and distribution of product molecules. For example, CO_2_R producing C_2_H_4_ would be well suited to leverage existing downstream infrastructure, including C_2_H_4_ pipelines and transportation hubs. In the base scenario, CO_2_ is captured and reduced at the point source and the product is transported to existing manufacturing facilities. In all other scenarios, a more distributed supply chain is depicted, where CO_2_ from various point sources is aggregated at existing petroleum storage terminals and reduced into a CO_2_R product. Petroleum terminals are more numerous and spatially diverse across the United States, making them possible candidates for a distributed CO_2_R network.

**Figure 2:**
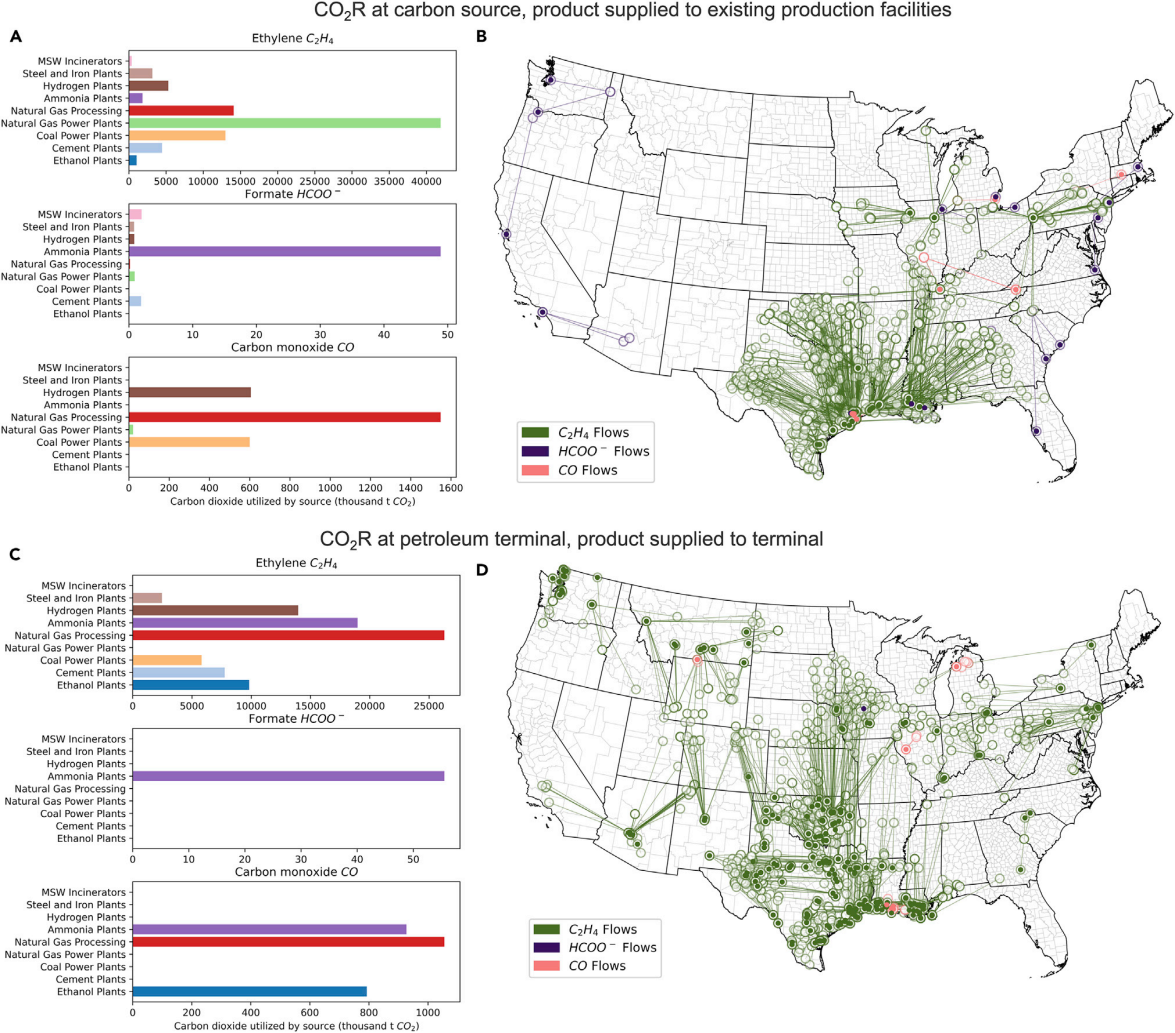
Inventories and CO_2_ supply mixes for C_2_H_4_, HCOO^−^, and CO for base scenario considering product allocation to existing infrastructure (A and B) and distributed scenario where products are allocated to petroleum terminals. (C and D). Lines in (B) and (D) connecting points represent flows of product from feedstock source to product sink, with filled points representing sink locations and hollow points represent sources of CO_2_.

The total number of CO_2_R systems needed to meet supply requirements vary drastically by product, with HCOO^−^ and CO transfers from sources to sinks equal to 17 and 20, respectively, compared to over 1,000 for C_2_H_4_ in the base scenario. This analysis suggests that a spatially and compositionally diverse distribution of CO_2_ sources must be used to provide sufficient feedstock for CO_2_R, with CO_2_ supply evolving from the base to distributed scenario (Figures 2A and 2B). Meeting demand for the three molecules requires utilization of CO_2_ from lower-concentration (and therefore more expensive) CO_2_ streams once supply from high-concentration streams is exhausted.

When designating existing infrastructure as sinks for CO_2_R products, the CO_2_ supply mix depends heavily on fossil fuel power plants and natural gas processing systems. C_2_H_4_ systems are heavily concentrated on the Gulf Coast, where most C_2_H_4_ manufacturing exists (Figure S1), and similar trends exist for HCOO^−^ and CO. The sources of CO_2_ utilized for each product tend to vary depending on the amount of CO_2_ needed. C_2_H_4_ and CO exhibit large market sizes and must therefore utilize CO_2_ from a variety of point sources, which leads to a more diverse supply. Because HCOO^−^ is produced in significantly lower quantities than other molecules, the model can allocate feedstocks from low-cost, high-concentration emissions.

Co-locating CO_2_R systems where target molecules are already produced or consumed at scale enables integration of CO_2_R technology into existing infrastructure and minimizes ancillary investments but doing so also limits potential CO_2_ feedstock streams to those closest to the existing infrastructure. When using existing chemical manufacturing facilities (Figure S1) as sink locations, flows are concentrated in the East and Southeast with existing petrochemical infrastructure (Figure 2B). The geographically concentrated sink locations assumed in the existing infrastructure scenario determine the types of CO_2_ sources leveraged in the model, with nearby sources of CO_2_ prioritized to minimize transportation costs.

In contrast to the concentrated sink locations in the base scenario, the distributed scenario depicts a flexible implementation of CO_2_R at scale by imagining the use of petroleum terminals as sink locations (Figures 2C and 2D). Petroleum terminals are already widely distributed throughout the country, allowing for greater flexibility in CO_2_ utilization (Figure S2) (HIFLD Open Data, 2021). Total domestic production for each product must still equal current market demand but can be allocated to any petroleum terminal rather than existing chemical manufacturing facilities as in the base scenario. Spatially distinct industrial electricity prices by county (NREL, 2018) are assigned for each petroleum terminal sink location, and a CO_2_R system is assumed to pay the industrial electricity price at the terminal (Figure S3). Low industrial electricity prices occur in locations with high variable renewable generation and in the Gulf Coast, where existing petrochemical processes exist. Processes are preferentially located in these regions, where low electricity prices reduce production costs for CO_2_R.

The entirety of the CO_2_ supply for HCOO^−^ and large portions of CO_2_ for C_2_H_4_ and CO is sourced from ammonia plants. Ammonia plants emissions exhibit the lowest carbon capture costs of the point sources considered (National Petroleum Council, 2019), and the spatial flexibility afforded when products can be allocated to petroleum terminals allows for full utilization of these low-cost feedstocks. C_2_H_4_ exhibits a more varied supply of CO_2_ in the distributed scenario but becomes less reliant on power sector point sources than in the base scenario. Across all products, the amount of CO_2_ captured from power plants decreases from the base to distributed scenario, and the utilization of smaller scale, lower cost high-concentration streams increases.

The distributed infrastructure scenario demonstrates that CO_2_R systems for chemical synthesis might not be deployed in areas already highly concentrated with existing industrial operations. Such a geographic shift has implications both for existing supply chains and environmental justice and equity. Because industrial manufacturing and other facilities that emit notable amounts of air pollution tend to disproportionately affect people in poverty and people of color (Elliott et al., 2004; Mikati et al., 2018; Lartey and Laughland, 2019; Carley and Konisky, 2020), a more geographically distributed chemical manufacturing sector with CO_2_R could move these processes away from these communities. While a shift of infrastructure away from marginalized communities might reduce health hazards for these communities, it can also negatively impact local economies that rely on the jobs that existing infrastructure provides. Understanding the specific costs and benefits to local communities that would arise from deployment of CO_2_R infrastructure is an important consideration for future work in this space.

A spatially distributed network for chemical synthesis also has the potential to create a more resilient supply chain. Recent research suggests that for multiple sectors, spatially distributed supply chains could be more economically efficient and resilient to disruptions, in addition to ensuring access to local feedstocks (Arai et al., 2009; Pettit et al., 2010; Becker et al., 2020). In the United States, hurricanes have significantly impacted petrochemical operations on the Gulf Coast in recent years, causing plant shutdowns, spills, and damages (Royal Dutch Shell, 2005; Euan, 2008; Forrester, 2020; Mufson and Fears, 2020). Distributed CO_2_R at scale could help create a more resilient supply chain for C_2_H_4_, HCOO^−^, and CO because single events are unlikely to impact as many producers. In other words, spatially distributed chemical synthesis suggests that supply chain disruptions from extreme weather and other external stressors could be less frequent and affect smaller portions of the overall supply chain.

### CO_2_ supply and the role of direct air capture

Annual United States CO_2_ emissions could provide sufficient feedstock in the near term for production of the three molecules analyzed here; however, eventual decarbonization of the U.S. power sector (Steinberg et al., 2017; Lawson, 2018; Victor et al., 2018; Luderer et al., 2019) is likely to reduce the number of point sources and overall amount of CO_2_ available, further constraining how and where CO_2_R might be developed. For example, if only CO_2_ point sources from ethanol plants are used in CO_2_R, this system would only be able to meet about 23% of annual ethylene production in the United States irrespective of spatial supply constraints. While certainly not a negligible amount, such a C_2_H_4_ supply chain would still be heavily reliant on fossil fuel feedstocks for the remainder of ethylene production. If CO_2_R were to be used in the production of basic chemicals and other industrial products in a future with a decarbonized power sector, DAC of CO_2_ might be needed to provide supplemental CO_2_. A similar need for DAC could emerge as the chemical industry attempts to reduce emissions. Such is the case with electrolysis-based ammonia production, which would reduce the availability of point source CO2 from ammonia plants (Hollevoet et al., 2020). Given the growing investment and development in DAC technologies, as well as the longevity of excess atmospheric CO_2_, this work considers DAC as a possible source of supplemental CO_2_.

Beyond decarbonization of the power sector, changes in existing industrial operations are likely to impact non-power sector CO_2_ streams that are available for use in CO_2_R systems. In the present work, we consider how changes in availability of CO_2_ from the power sector impact CO_2_R deployment, creating opportunities for DAC. Changes in non-power sector CO_2_ sources are not depicted here because the pathways and timelines for decarbonization of these processes are more uncertain than the power sector; however, the importance of changing industrial processes and the resulting impacts on CO_2_R deployment should be noted.

To analyze how DAC might support CO_2_R-based supply chains, a series of model scenarios that vary the supply of point source CO_2_ were developed to depict how the dynamics of CO_2_R might change when DAC CO_2_ is available as a feedstock source. These scenarios adopt the same assumptions as the distributed scenario, and assume DAC takes place on-site at a petroleum terminal. For distributed scenarios with DAC as a potential source of CO_2_, it is assumed that fossil fuel power generation is unavailable for capture, because of either excessive retrofitting costs or plant retirement.

**Figure 3:**
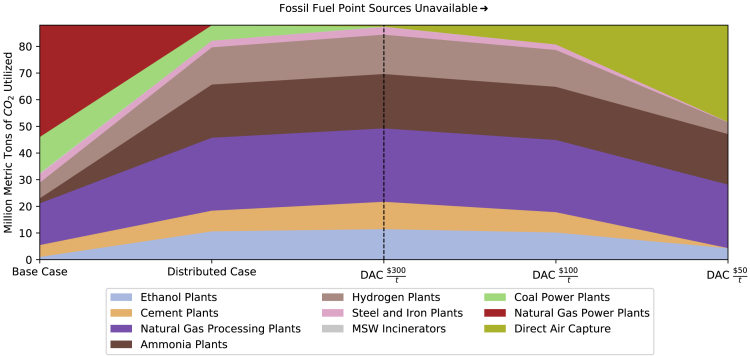
CO_2_R feedstock supply mix under different scenarios, including existing and distributed sink infrastructure, with and with varying DAC costs Scenarios shown from the dashed line to the right have fossil fuel point sources excluded from the model. See Table 1 for documentation of assumptions specific to the scenarios shown here.

Utilization of CO_2_ from fossil fuel point sources decreases from the base to distributed scenario as discussed prior, with capture of smaller and more spatially distributed point sources such as ammonia and ethanol plants increasing because of the greater spatial flexibility depicted in the distributed scenario (Figure 3). Natural gas and coal power plants are more expensive for carbon capture than other point sources, and their use decreases in the distributed scenario.

DAC serves as a source of CO_2_ only when its cost is competitive with carbon capture of CO_2_ from existing point sources. At a cost of $300/t CO_2_ captured, DAC is not developed because most existing point sources are more economical to utilize. The amount of DAC utilized evolves depending on how economically competitive DAC is with carbon capture from existing CO_2_ point sources. As DAC decreases in cost, more expensive carbon capture systems are avoided in favor of lower-cost DAC.

At a cost of $50/t CO_2_ captured, DAC generates about 40% of the CO_2_ utilized in CO_2_R. From a purely economic perspective, DAC appears to represent a significant opportunity for use in CO_2_R if cost competitive with capture and purification of point source streams. Recent research has critically analyzed the potential for significant scale-up in DAC systems, and it has identified the significant energy and resource demands inherent in scale-up for these systems (Realmonte et al., 2019, 2020; Chatterjee and Huang, 2020). Although resource intensity estimates are beyond the scope of this work, they merit consideration as potential constraints on the total capacity of DAC deployed.

**Figure 4:**
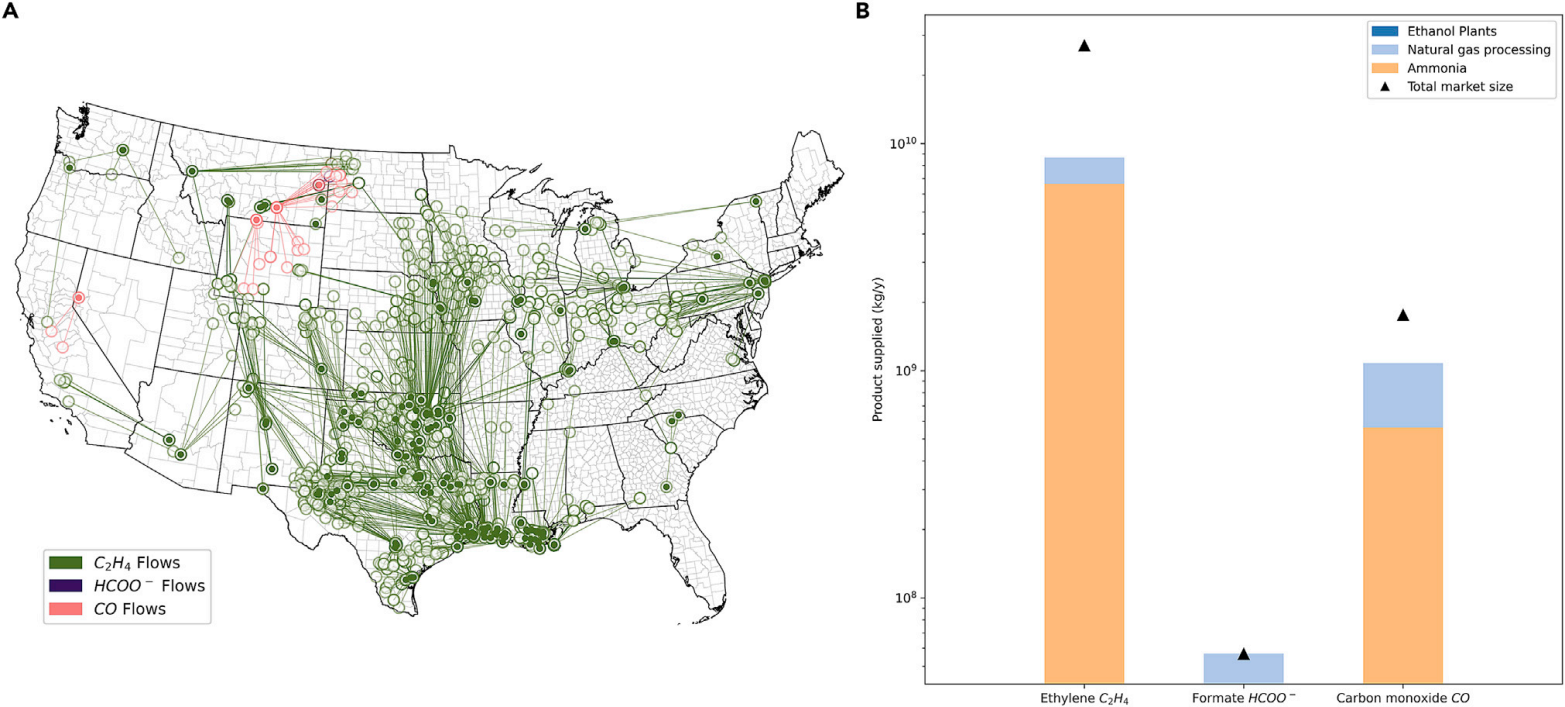
Optimization model results for high-concentration feedstock scenario (A) Spatial distribution of sinks (filled points) and sources (empty points). (B) Product supplied via CO_2_R by source compared against current market size, illustrating the need for supplemental sources of CO_2_ when the model is constrained to only using high-concentration streams from ethanol, ammonia, and natural gas processing facilities.

The high-concentration feedstock scenario was designed to depict the potential impact that utilization of only high-concentration CO_2_ sources could have toward meeting total market supplies in the United States. Ethanol plants, natural gas processing facilities, and ammonia plants are considered high-concentration sources for the purpose of this scenario, although other smaller scale sources might exist. While using only these sources, substantial portions of the total markets for these products can be supplied via CO_2_R, utilizing a spatially distributed network of sources (Figure 4). These point sources can be captured and purified to supply CO_2_R systems at the lowest cost, making them attractive near-term opportunities for pilot scale system development. For development of a complete CO_2_R-based supply chain for the three products considered here, carbon capture of lower concentration CO_2_ sources is needed, and/or DAC.

In the high-concentration feedstock scenario, only the market size for formate was met with CO_2_R, with insufficient CO_2_ to meet demand for ethylene and carbon monoxide (Figure 4B). With respect to the entire organic chemical sector, the three products considered here are an insufficient representation of the total CO_2_ that would be required for a complete shift to CO_2_R-based supply chains. A shift toward a decarbonized power sector is likely to significantly reduce the amount of CO_2_ available for these systems, further underscoring the importance of DAC as a source of CO_2_. This analysis focuses solely on the potential for CO_2_R to supply products in the organic chemicals sector; however, further applications including transportation fuels and industrial operations could also increase the demand for CO_2_R products and thus the need for increased DAC.

### Energy demand for a CO_2_R-based chemical industry

Assuming such a significant deployment of CO_2_R necessitates consideration of the logistical feasibility and resource intensity of developing these systems. Conventional synthesis for the products considered here generally involves using natural gas as a feedstock at large-scale facilities. CO_2_R does not depend on natural gas, but it does consume electricity and water in the reduction of CO_2_, and mature processes are likely to operate at smaller scales and in more spatially distributed supply chains.

**Figure 5:**
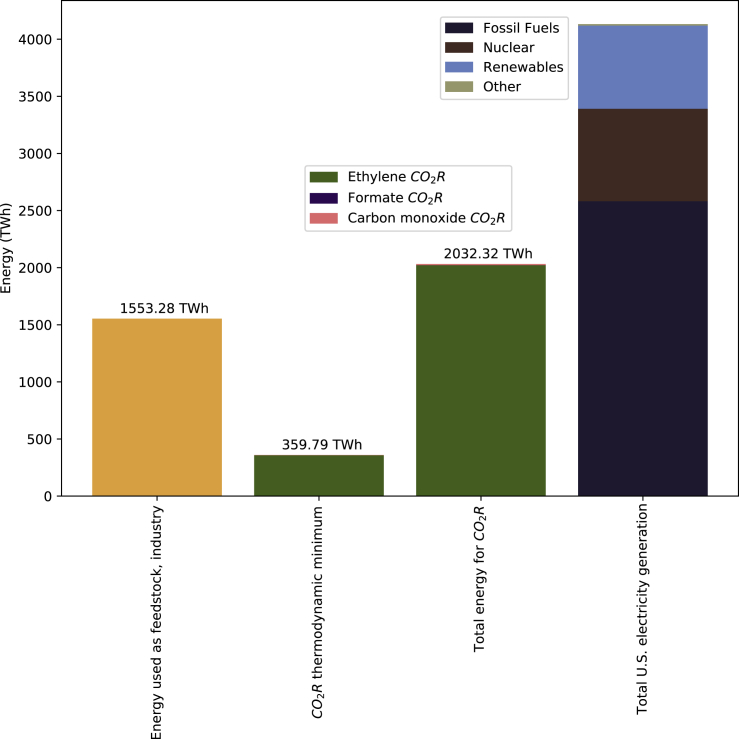
Energy demands for CO_2_R compared to feedstock energy consumption in industry and total electricity generation in the United States

Based on the current required and whole-cell potentials described in literature (Table S1), Figure 5 estimates the energy required to synthesize the three chemicals at current market sizes. C_2_H_4_ comprises most of the total electricity required because 12 electrons are required per molecule synthesized for C_2_H_4_ versus two for HCOO^−^ and CO, and C_2_H_4_ also has a significantly larger market size than HCOO^−^ and CO, and therefore more current (and in turn energy) is required to meet current market demand (Figure 5).

The total energy required to synthesize the products considered here via CO_2_R exceeds 1,900 TWh and would comprise approximately 46% of annual electricity generation in the United States (Figure 5) (U.S. EIA, 2021). To further contextualize the energy requirements depicted here, total industrial energy consumed as feedstocks (hydrocarbon gas liquids, natural gas, coal, and coke) in the United States totals 1,550 TWh (U.S. EIA, 2014), or slightly less than the total CO_2_R energy consumption shown, which is only for production of C_2_H_4_, HCOO^−^, and CO. The energy intensity for current production pathways of C_2_H_4_ is estimated at 26 GJ/t (Worrel et al., 2000). Assuming a domestic market size of 27.12 million t per year, energy required for today’s C_2_H_4_ production pathways is roughly 195 TWh.

The energy consumption for CO_2_R shown here is solely that used for the CO_2_R reaction itself and does not account for energy consumed in point source carbon capture, CO_2_R reactor outlet separations, and DAC, and it is therefore an underestimate of total energy required across all systems. For context, commercial DAC systems require approximately 5 GJ thermal energy/t CO_2_ in addition to smaller electrical energy requirements (McQueen et al., 2020). If DAC systems are deployed at large scales to support CO_2_R, the resulting energy demands could be significant.

Of the approximately 2,000 TWh estimated, about 360 TWh are required for the standard reduction potential (the thermodynamic, reversible energy required) for each reaction (Table S3). The remainder of the energy represents overpotentials attributable to various components in the CO_2_R system, reduction of which has been discussed in recent reviews and experimental work (Li and Kanan, 2012; Jhong et al., 2013; Sun et al., 2017). Much of this energy is dissipated as heat via ionic Joule heating through the membrane and imperfect electronic contact of the metal and carbon phases. Some of this energy dissipated as heat could be reclaimed through thoughtful balance of plant design, increasing the efficiency of the plant. Thus, we expect that future advances in CO_2_R system design will reduce the overpotentials of these systems and reduce the total electricity consumed per kg of product.

Life cycle assessments of electrolysis and other power-to-gas technologies find that the carbon footprint of power supplied to the process is a key determinant of the carbon intensity of the product (Bhandari et al., 2014; Sadok et al., 2020), which underscores the importance of powering CO_2_R with renewable electricity. Powering CO_2_R entirely with renewable electricity implies a significant investment in renewable energy generation capacity to power these systems. New renewable generation at the scales depicted here is significantly more than is currently generated in the United States (Figure 5), and the new generation would represent a significant portion of total energy demand in the United States even without including energy requirements for carbon capture, product purification, and/or DAC. Although the availability and feasibility of developing additional renewable generation to meet the demands of CO_2_R is beyond the scope of the quantitative evaluation presented, it is an important consideration of the feasibility of developing CO_2_R and should be considered in future work.

## Discussion

This work analyzed several key questions not yet addressed in existing CO_2_R research: 1) what CO_2_ point sources are best for carbon capture and as sources of feedstock for CO_2_R, 2) how the dynamics of CO_2_ supply shift as CO_2_ point sources change and across a range of DAC costs, 3) the spatial dynamics of CO_2_R and how a network of CO_2_R facilities can be spatially optimized, and 4) the electricity demands for CO_2_R at scale in the context of current energy systems.

From both a cost and a system performance perspective, it is preferable to use high-concentration waste CO_2_ streams available from facilities such as ethanol and ammonia plants than low concentration CO_2_ streams. However, limiting CO_2_R to only use such sources would severely impact the supply of feedstock available for use. High-concentration CO_2_ streams represent just 2.6% of the total CO_2_ supply. Addressing this shortage in supply requires either capture and purification of low-concentration CO_2_ streams or development of CO_2_R reactors that are tolerant to flows with low CO_2_ concentrations.

The inventory of large stationary CO_2_ point sources in the United States is not static. Variability is especially prevalent in the power sector, which comprises the majority of the CO_2_ feedstock streams considered in this analysis. When large CO_2_ sources such as coal power plants are taken offline, the total supply of CO_2_ feedstock decreases and thereby reduces total feedstock available for CO_2_R. DAC could be needed to supplement CO_2_ supplied solely from high concentration, non-power sector point sources, depending on future demand for carbon-based products. Although this analysis only considered three products from the chemical and petrochemical manufacturing industries, DAC might be needed to provide enough CO_2_ for production of C_2_H_4_, HCOO^−^, and CO alone via CO_2_R when fossil fuel point sources and other associated low-concentration CO_2_ streams are unavailable. The cost of DAC relative to the cost of carbon capture from existing point sources determines the favorability of implementing DAC versus carbon capture of point source CO_2_.

There are significant spatial considerations when deploying CO_2_R at scale for both CO_2_ and electricity supply. If CO_2_R is allowed to utilize spatially distributed sources of CO_2_, CO_2_R is likely to be sited in places that provide access to low-cost electricity and CO_2_. This analysis depicts a system of geographically distributed CO_2_R plants, with increased dispersal of infrastructure relative to existing supply chains. A dispersed system could prove more resilient to impacts from natural disasters like those that have affected existing manufacturing infrastructure in recent years. DAC coupled with CO_2_R further enhances the spatial flexibility of these systems, thus eliminating constraints associated with fixed CO_2_ point sources.

Nation-scale CO_2_R deployment was analyzed with national datasets and an optimization framework. The amount of energy consumed is determined by the throughput of the reactor and the electrical potential of the reactor. At current system potentials, this work estimates that producing the three molecules considered here at their current market sizes would consume about 50% of the electricity that is generated annually in the United States, although we expect advances in reactor design and materials to reduce overpotentials and increase the energy efficiency of the process. CO_2_R coupled with renewable energy represents a significant opportunity for implementation of flexible loads that can utilize excess renewable power.

### Limitations of the study

This work uses numerical optimization to model possible pathways toward integrating electrolysis of point source and direct air capture (DAC) CO_2_ into existing organic chemical supply chains. The demand side (organic chemicals) and feedstock side (point source and DAC CO_2_) are subject to evolve because of policy drivers and concurrent changes in other sectors that are not considered in this work. Additionally, project resources only permitted modeling of three possible products from CO_2_ reduction; however, many other possible products exist. These other products could be produced directly in a single-step CO_2_R system or could be produced from multi-stage processes. Depending on the size of the market and selling prices for these products, they could create competition and further increase demand for point source and atmospheric CO_2_. Finally, advances in the performance and design of CO_2_R systems might create near-term opportunities for deployment that are not obvious given today’s state of this technology.

## STAR★Methods

### Key resources table

**Table:** 

REAGENT or RESOURCE	SOURCE	IDENTIFIER
**Deposited data**		

Point source CO_2_ emission data	EPA FLIGHT	https://ghgdata.epa.gov/ghgp/main.do
CO_2_ flue gas capture and purification cost estimates	National Petroleum Council: Meeting the Dual Challenge A Roadmap to At-Scale Deployment of Carbon Capture, Use, and Storage	https://dualchallenge.npc.org/downloads.php

**Software and algorithms**		

Spatial CO_2_R optimization model	Original work	https://github.com/NREL/CO2ROpt https://doi.org/10.5281/zenodo.6385504

### Resource availability

#### Lead contact

Further information and requests for resources should be directed to and can be fulfilled by the lead contact, Alex Badgett: alex.badgett@nrel.gov.

#### Materials availability

This study did not generate unique reagents.

### Method details

Most CO_2_R reactors have a cathode where CO_2_ is reduced and an anode where oxygen is generated (evolved). The oxygen evolution reaction (OER) can be written to reflect the pH of the media it takes place in.

(Equation 1) $$Acidic\;2{\text{H}}_{2}\text{O}\to {\text{O}}_{2}+4{\text{H}}^{+}+4{\text{e}}^{-}$$

(Equation 2) $$Alkaline\;4{\text{OH}}^{-}\to {\text{O}}_{2}+{\text{H}}_{2}\text{O}+4{\text{e}}^{-}$$

This work considers CO_2_R to three specific products: ethylene (C_2_H_4_), formate (HCOO^−^), and carbon monoxide (CO). Anode, cathode, and overall reactions to form these products are shown in Equations 3, 4, 5, 6, 7, 8, 9, 10, and 11. All reactions are assumed to take place in alkaline media. The optimization model developed in this work incorporates current performance metrics for CO_2_R systems, assuming constant performance for these systems. The performance of a CO_2_R determines the amount of product per kWh of electricity supplied to the system, and advances in performance are expected to increase the rate of product synthesis from these systems. Scenario and sensitivity analysis, while beyond the scope of the present analysis, is an important opportunity for future work to address.

(Equation 3) $${\text{ C}}_{2}{\text{H}}_{4}{\text{CO}}_{2}R\;2{\text{CO}}_{2}+8{\text{H}}_{2}\text{O }+12{\text{e}}^{-}\to {\text{ C}}_{2}{\text{H}}_{4}+12{\text{OH}}^{-}$$

(Equation 4) $${\text{ C}}_{2}{\text{H}}_{4}\;\text{OE}R\;12{\text{OH}}^{-}\to 3{\text{O}}_{2}+6{\text{H}}_{2}\text{O}+12{\text{e}}^{-}$$

(Equation 5) $$Net\;reaction\;2{\text{CO}}_{2}+8{\text{H}}_{2}\text{O}\to {\text{C}}_{2}{\text{H}}_{4}+3{\text{O}}_{2}+6{\text{H}}_{2}\text{O}$$

(Equation 6) $${\text{HCOO}}^{-}{\text{CO}}_{2}R\;{\text{CO}}_{2}+{\text{ H}}_{2}\text{O }+2{\text{e}}^{-}\to {\text{ HCOO}}^{-}+{\text{OH}}^{-}$$

(Equation 7) $${\text{HCOO}}^{-}\;\text{O}ER\;2{\text{OH}}^{-}\to \frac{1}{2}{\text{O}}_{2}+{\text{H}}_{2}\text{O}+2{\text{e}}^{-}$$

(Equation 8) $$Net\;reaction\;{\text{CO}}_{2}+2{\text{H}}_{2}\text{O}\to \text{HCOOH}+\frac{1}{2}{\text{O}}_{2}+{\text{H}}_{2}\text{O}$$

(Equation 9) $$\text{CO}{\text{CO}}_{2}R\;{\text{CO}}_{2}+{\text{ H}}_{2}\text{O }+2{\text{e}}^{-}\to \text{ CO}+2{\text{OH}}^{-}$$

(Equation 10) $$\text{CO}\;\text{O}ER\;2{\text{OH}}^{-}\to \frac{1}{2}{\text{O}}_{2}+{\text{H}}_{2}\text{O}+2{\text{e}}^{-}$$

(Equation 11) $$Net\;reaction\;{\text{CO}}_{2}+{\text{H}}_{2}\text{O}\to \text{CO}+\frac{1}{2}{\text{O}}_{2}+{\text{H}}_{2}\text{O}$$

Recent studies have focused on advances in the electrochemical CO_2_R system itself under the assumption that CO_2_ feedstock streams are without impurities. Although notable amounts of high-concentration CO_2_ streams exist across the United States, lower-concentration CO_2_ sources must be utilized to provide sufficient carbon for operating CO_2_R at scale. In this work, we assume CO_2_ point source emissions are captured and purified and used as the carbon source for CO_2_R.

Point-by-point annual CO_2_ emissions equaling 2.13 billion metric tons annually (Ely and Rock, 2014; U.S. EIA, 2020b) and the cost of capturing and purifying various CO_2_ streams (Bains et al., 2017; National Petroleum Council, 2019) as a function of facility type are used to model feedstock allocation for CO_2_R (Table S2). According to the United States Environmental Protection Agency (EPA) Inventory of U.S. Greenhouse Gas Emissions and Sinks, large facilities emit approximately 2.71 billion metric tons of CO_2_ annually, indicating our model considers approximately 79% of CO_2_ emitted from large point sources. This 21% discrepancy results from our model not considering smaller point sources in the EPA dataset (U.S. EPA, 2019), such as universities, food processing, other manufacturing, and point sources without sufficient CO_2_ capture cost information to be included in our model. CO_2_ point sources vary by concentration and composition, and how the carbon emitted from them is accounted for in greenhouse gas inventories. The optimization model used in this work only considers economic and not environmental factors for utilizing different CO_2_ sources, and solely optimizes CO_2_ supply at the lowest cost.

We define a net present value (NPV) function (Equation 12), which estimates the NPV of a CO_2_R system as a function of cost and revenue streams for a CO_2_ point source *i*, product sink *j*, and CO_2_R product *k* in units of dollars per year. $x_{i,j,k}$ represents the flow of a given CO_2_R product *k* from a CO_2_ point source *i* to product consumer *j* in kilograms per year (kg/year). In the following text, we discuss each term of this equation in detail.

(Equation 12) $$\text{NPV}=v_{k}x_{i,j,k}-e_{i,k}x_{i,k}-D_{i,j}t_{i,j,k}x_{i,j,k}-s_{i,k}x_{i,k}-c_{i}x_{i,j,k}$$

The first term in Equation 12 represents the value of the product from the CO_2_R system, where $v_{k}$ is the product value in $/kg. The value of this term is influenced by the amount of product and the product market price. High market prices, such as those for HCOO^−^ increase the revenue stream and overall NPV.

In the second term of Equation 12, $e_{i,k}$ represents the cost of electrons required to reduce CO_2_ to product *k* in dollars per kg. $e_{i,k}$ is estimated using Faradays Law of electrolysis (Equation 13) (Jouny et al., 2018). $E_{k}$ is the applied cell potential in volts, $q_{k}$ is the number of electrons required per molecule of species *k* (Jouny et al., 2018), $P_{i}$ is the regional industrial electricity price in dollars per kWh (NREL, 2018), F is Faraday’s constant (96,485C/mol), $mw_{k}$ is the molar mass of species *k*, and $FE_{k}$ is the faradaic efficiency of CO2R for species *k* (Grim et al., 2019).

(Equation 13) $$e_{i,k}=\frac{E_{k}q_{k}P_{i}F}{mw_{k}FE_{k}}$$

The third term of Equation 12 estimates the transportation cost of moving CO_2_ from CO_2_ point source *i* to a product consumer *j*. In this term, $D_{i,j}$ represents the city block distance between a CO_2_ point source *i* and product consumer *j* in meters. The city block distance formula is used to approximate the distance between sink and source points (Equation 14). In reality, the distance between two points is a function of the transportation mode used, and city-block distance is used as an approximate distance relationship in this case. The cost per kg per kilometer to transport each ton CO_2_ is represented by the variable $t_{i,j,k}$ (Doctor et al., 2018).

(Equation 14) $$D_{i,j}=\left|i_{x}-j_{x}\right|+|i_{y}-j_{y}|$$

In the fourth term, the variable $s_{i,k}$ represents the capital and operating costs (excluding electricity costs, which are accounted for in the second term of Equation 12) of the electrochemical reactor. To estimate the balance-of-plant cost of these systems, we adopt and modify a technoeconomic model by Jouny et al. (2018) to approximate separation system costs at different scales (Jouny et al., 2018). Jouny et al. assume a simplified outlet separations process and are likely an underestimate of costs of outlet separation for CO_2_R systems, which are likely to produce multiple products that need to be separated from unreacted CO_2_ in the outlet stream. Also included in these estimates are costs for the electrolyzer stack and downstream separations train. Capital costs for CO_2_R electrolyzers are assumed to be equal to $10,000/m^2^. Active area of electrolyzer required is determined based on the amps required to produce product $x_{i,j,k}$, the current density, and faradaic efficiency of the system (Table S1) (Ma et al., 2016; Grim et al., 2019; Chen et al., 2020; García de Arquer et al., 2020).

In the last term, $c_{i}$ represents the cost of CO_2_ feedstock, normalized to dollars per kg of product *k* based on the carbon content of the product. Operation of a CO_2_R system requires a high-concentration flow of CO_2_. Because many of the CO_2_ point sources considered in this work generate low-concentration streams (∼10% CO_2_), we include the cost of purifying these streams (National Petroleum Council, 2019). Carbon capture costs are differentiated between CO_2_ point sources and generally depend on the initial concentration of CO_2_ in the outlet stream.

Optimization of the NPV function (Equation 12) is subject to four constraints. These assumptions constrain CO_2_R systems to realistic scales and ensure nearby sources of CO_2_ are allocated by the model. Our model seeks to maximize the objective function, Equation 12, subject to the constraints (Equation 15).

(Equation 15) $$max\;NPV\;s.t.\{\begin{array}{l} \begin{array}{l} \sum_{i,k}x_{i,k}\leq a_{i}\\ \sum_{i,j}x_{i,j}\leq d_{j}\\ \sum_{i,k}e_{i,k}\leq 500 \end{array}\\ D_{i,j}<1,000 \end{array}$$

The first constraint represents the mass balance at each CO_2_ point source. Total flows from a point source *i* for all CO_2_R products *k* must be less than or equal to the total feedstock available at that point ($a_{i}$). Because the products modeled have different molecular compositions, flows and CO_2_ feedstocks are normalized to a kilograms-carbon basis.

The second constraint ensures total inflows to a product consumer point *j* that consumes a product species *k* do not exceed the total demand at that point across all point sources *i*. In the base, distributed, and DAC scenarios ∑a>∑d, indicating that the system is not feedstock constrained. In the limited feedstock scenario, the system is constrained by the amount of CO_2_, and ∑a<∑d.

The third constraint requires that power consumed in a CO_2_R reactor not exceed 500 MW. This constraint ensures the size of a CO_2_R plant is kept within a realistic range. For reference, the largest proton exchange membrane water electrolysis system planned for construction in Germany has a power consumption of 100 MW (Freist, 2019; FuelCellsWorks, 2019).

The final constraint in Equation 15 represents a limitation on distance in kilometers (1,000) between CO_2_ point source and product sink. This constraint is chosen to confirm the model prioritizes nearby sources of CO_2_ while ensuring sufficient CO_2_ is available to meet the demand at all sink points. This distance constraint minimizes the development of transportation infrastructure for moving CO_2_R products from source to sink locations.

To estimate a maximized solution to Equation 12, this work uses the JuMP optimization framework within the Julia programming language to develop solutions to the linear optimization problem presented (Bezanson et al., 2017; Dunning et al., 2017). JuMP uses the GNU linear programming kit, which implements a revised simplex method to minimize the relevant variables.

Several different product allocation scenarios are considered. Most of the molecules considered here are produced by a handful of industrial sites in the United States. A literature search was conducted to identify the location and capacity of these sites.

For C_2_H_4_, products are allocated to ethane crackers, which use heat to crack ethane into C_2_H_4_. Most ethane crackers exist along the Gulf Coast, close to the petrochemical infrastructure they rely on for ethane feedstocks (DOE, 2018).

It is assumed HCOO^−^ is produced via CO_2_R, with a subsequent acid titration step to produce formic acid (HCOOH). We assume the added cost of the acid titration step is negligible with respect to overall costs. We were only able to identify one plant producing formic acid in the United States, located in the Gulf Coast. About 6 million kg per year of formic acid is imported through various ports, which are also designated as sinks for formic acid (United States International Trade Commission, 2020).

It is assumed the carbon monoxide produced from CO_2_R would be used in acetic acid production. Equation 16 shows the methanol carbonylation reaction to form acetic acid, using carbon monoxide as an input to the process (Kalck et al., 2020). Using stoichiometric ratios of the reaction, we estimate consumption of CO to be 0.46 kg CO per kg acetic acid. Acetic acid facility production rates are used to back-calculate the amount of CO required.

(Equation 16) $${\text{CH}}_{3}\text{OH}+\text{CO}\to {\text{CH}}_{3}\text{COOH}$$

We assume a 50% single pass conversion efficiency of CO_2_ in CO_2_R reactors. Low single pass conversion significantly increases downstream outlet separations costs by increasing the magnitude of outlet flows and diluting product streams (Jeng and Jiao, 2020). Based on current performance of these systems at benchtop scales, 50% is an optimistic estimate of single pass conversion. Reported values can range from 0.3% to 68%, and this topic has only recently been examined (Ripatti et al., 2019; Ross et al., 2019).

As renewable power sources are temporally variable sources of electricity, we account for the purchase of battery energy storage to deliver consistent power to a CO_2_R facility. Battery costs per unit capacity of $1,500/kW are used to represent an average cost rate from short-duration (<0.5 h) to medium-duration (2-4 h) energy storage (U.S. EIA, 2020a). We assume the total battery power purchased is equal to the total power rating of the CO_2_R system.

The inventory of large CO_2_ point sources in the United States is not static, and the eventual decarbonization of the U.S. power sector (Steinberg et al., 2017; Lawson, 2018; Victor et al., 2018; Luderer et al., 2019) will impact the availability of CO_2_ streams and make it more difficult to source CO_2_ for production of organic chemicals via CO_2_R. Although the exact timeline and degree of power sector decarbonization is unknown, we assume 1) not all fossil fuel generation CO_2_ sources are feasible for carbon capture, because either retrofitting costs are excessively high or the generator has been closed and 2) electricity prices remain equal to current 2018 rates.

Though recent work has reviewed the technical side of CO_2_R and the application of large-scale DAC systems for geological storage, it remains unclear how DAC would realistically respond to the large demand for CO_2_ from CO_2_R process at a nationwide scale (Jouny et al., 2018; Fasihi et al., 2019; McQueen et al., 2020). Unlike the CO_2_ source streams from emissions point sources, there are no fixed DAC locations in the United States. A DAC plant that is co-located at a chemical sink would ideally minimize transportation costs and allow for integration into a closed-loop system, such as the pairing of DAC with CO_2_R (Keith et al., 2018). For DAC locations, it is assumed each plant can supply up to 100,000 t of CO_2_ per year for downstream use in the electrochemical system. This estimate is based on DAC production from private companies with an operational DAC plant. A DAC plant proposed by Carbon Engineering would produce a stream of 1 million t of CO_2_/year (Carbon Engineering, 2020), placing our assumption on the size of DAC facilities well within realistic limits. Also for this study, 100,000 t/year is chosen because it is the minimum size for a DAC plant to qualify for Section 45Q of the U.S. tax code (U.S. House of Representatives, 2018).

Cost estimates for DAC are highly variable and have ranged from over $1,000 to less than $50/t CO_2_ captured (Ishimoto et al., 2017; Keith et al., 2018; Fasihi et al., 2019). For this work, we analyze DAC costs ranging from $300 to $50/t CO_2_ captured.

The molar and mass rate of water consumed for all three products is estimated with Equations 17 and 18, where $s_{h}$ is the stoichiometric coefficient equaling 2, 3/2, 1/2 for C_2_H_4_, and CO, respectively, from Equations 3, 4, 5, 6, 7, 8, 9, 10, and 11.

(Equation 17) $${\dot{n}}_{water,consumed}=-s_{h}{\dot{n}}_{product,generated}$$

(Equation 18) $${\dot{m}}_{\text{water},\text{consumed}}=s_{h}\frac{MM_{\text{water}}}{MM_{\text{product}}}{\dot{m}}_{\text{product}}$$

For calculations of standard reduction potentials, the following is assumed:

• Boiling points for C_2_H_4_/HCOO^−^/CO ≈ -100°C/+100°C/-191°C.
• Products generated are gas/liquid/gas for C_2_H_4_/HCOO^−^/CO respectively.
• The reactor is operated at 25°C.
• The anode is fed aqueous electrolyte and is therefore always liquid-saturated.
• Because of the reactor temperature and water balance in a reactor fed with water vapor-saturated CO_2_, the high heating value (HHV) for water should be used in thermodynamic calculations. This assumes water generated in the cathode is not vaporized, and thus, the latent heat energy is not subtracted from the thermodynamic potential.

The standard reduction potentials for the HHV are determined from Equations 19 and 20. θ denotes a standard state property (Table S3).

(Equation 19) $$\text{Δ}G_{f}^{\theta }=\text{Δ}H_{f}^{\theta }-T_{std}\text{Δ}S_{f}^{\theta }$$

(Equation 20) $$E^{\theta }=-\frac{\sum \text{Δ}G^{\theta }}{nF}$$

Standard reduction potentials for the three reactions are 1.15 V, 1.39 V and 1.33 V for C_2_H_4_, HCOO^-^, CO respectively.

## Data Availability

• Optimization model code and input data have been deposited at a GitHub repository and are publicly available as of the date of publication. Accession links are listed in the key resources table. • All original code has been deposited at Zenodo and is publicly available as of the date of publication. DOIs are listed in the key resources table. • Any additional information required to reanalyze the data reported in this paper is available from the lead contact upon request.
