# Supplementary material for: Optimizing utilization of point source and atmospheric carbon dioxide as a feedstock in electrochemical CO2 reduction
Source: iScience. 2022 Apr 18;25(5):104270. doi: 10.1016/j.isci.2022.104270 (PMC9065307; doi:10.1016/j.isci.2022.104270)
Supplement: Document S1. Figures S1–S3 and Tables S1–S3 [file mmc1.pdf]

iScience, Volume *25*

Supplemental information

Optimizing utilization of point source and atmospheric carbon dioxide as a feedstock in electrochemical CO_2 reduction

Alex Badgett, Alison Feise, and Andrew Star

Table S1. Operational parameters for CO2R systems, related to STAR methods.

	Ethylene	Formate	Carbon Monoxide	Units
Whole cell potential	3.9 (García de Arquer *et al.*, 2020)	5.8 (Chen *et al.*, 2020)	3 (Ma *et al.*, 2016)	V
Current density	1.55 (García de Arquer *et al.*, 2020)	0.5 (Chen *et al.*, 2020)	0.35 (Ma *et al.*, 2016)	A/cm^2
Faradaic efficiency	0.6 (García de Arquer *et al.*, 2020)	0.93 (Chen *et al.*, 2020)	0.95 (Ma *et al.*, 2016)	%
Water consumption	1.28[a]	0.585[a]	0.32[a]	kg water/kg product
Market value	0.71(Grim *et al.*, 2019)	0.66 (Grim *et al.*, 2019)	0.18 (Grim *et al.*, 2019)	$/kg
Single pass conversion	0.5[b]	0.5[b]	0.5[b]	%
Modeled market size	27.12[c]	0.056[c,d]	1.76[c]	Billion kg/year
Electrons/molecule (*z*)	12	2	2	e^- / molecule

[a] Calculated from stochiometric ratios of Eqns. 5, 8, and 11

[b] It is assumed that at-scale CO_2R systems will exhibit higher single pass conversion efficiency (50%) than current experimental systems, but this value is an optimistic representation of most current systems.

[c] Determined from online search of existing chemical manufacturing facilities and associated annual capacities

[d] Determined from imports from United States International Trade Commission trade data

Table S2. Summary of carbon capture costs for CO_2 point sources and total emissions, related to STAR methods.

Facility Type	Carbon Capture Cost [$/t CO2]	Total Annual Emissions [MMt CO2]
Ammonia plants	25	35.72
Cement plants	80	66.84
Large coal power plants	103	912.68
Medium coal power plants	146	111.56
Small coal power plants	217	143.21
Ethanol plants	29	19.65
Hydrogen plants[1]	75	45.59
Municipal solid waste incinerators	140[2]	13.77
Large natural gas power plants	117	313.93
Medium natural gas power plants	157	117.25
Small natural gas power plants	235	229.84
Steel and iron plants	75	75.74
Natural gas processing plants	29	47.24

[1] This work assumes that hydrogen production plants use pressure swing adsorption (PSA) to purify hydrogen. PSA purification leads to CO_2 concentrations of about 45%, while other technologies such as CO_2 scrubbing can produce high concentration CO_2 streams that could be available at lower costs. (Bains, Psarras and Wilcox, 2017)

[2] Carbon capture costs are assumed to be comparable to those of a power plant and are approximated to be near the average carbon capture cost of a large and medium natural gas power plant.

Table S3. Table of enthalpies and entropies of formation for key compounds at 1 atm and 298˚K, related to STAR methods.

**Compound**	**Standard Enthalpy of Formation (J/mol)**	**Standard Entropy of Formation (J/mol*K)**	**Standard Gibbs energy of Formation (J/mol)**
CO_2	-393520	214	-457324.1
H_2O	-285830	70	-306700.5
C_2H_4	52470	219	-12824.85
HCOOH	-425090	128.4	-463372.46
CO	-110530	197	-169265.55
O_2	0	205	-61120.75

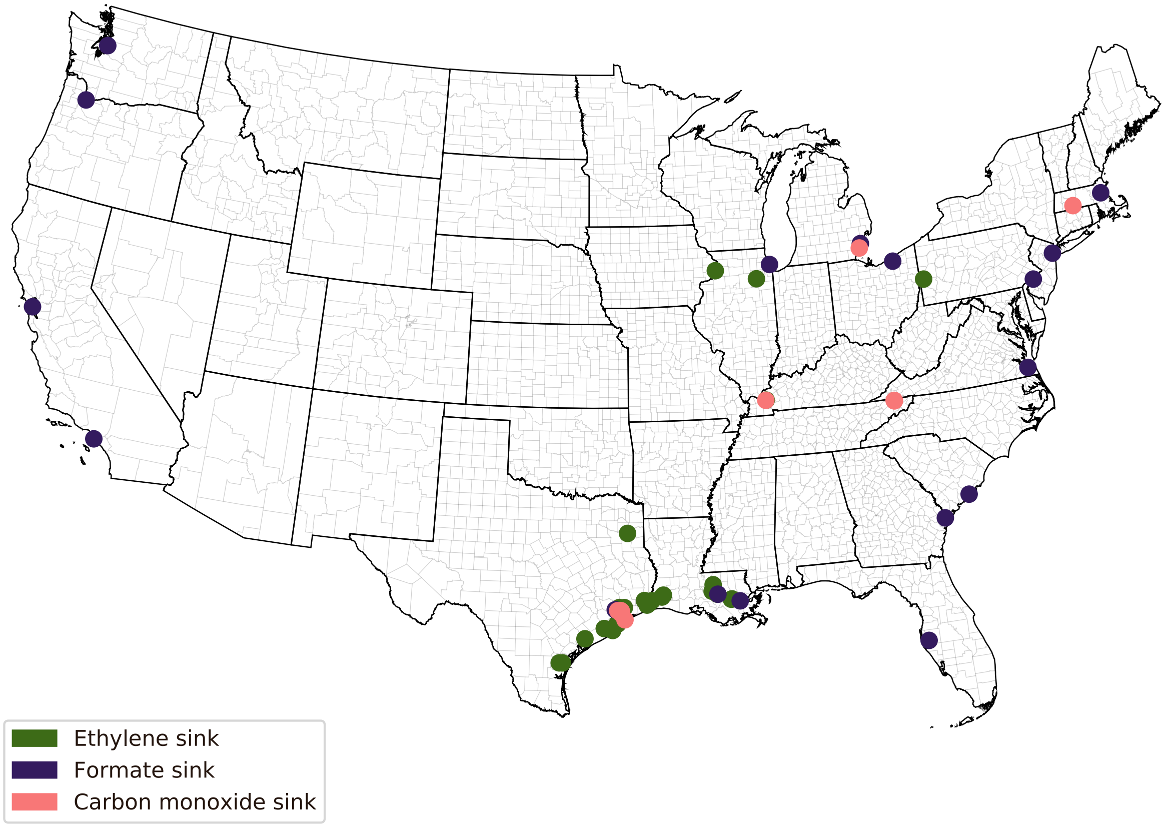

Figure S1. Map of base scenario product sink locations, related to STAR methods and Base Scenario.

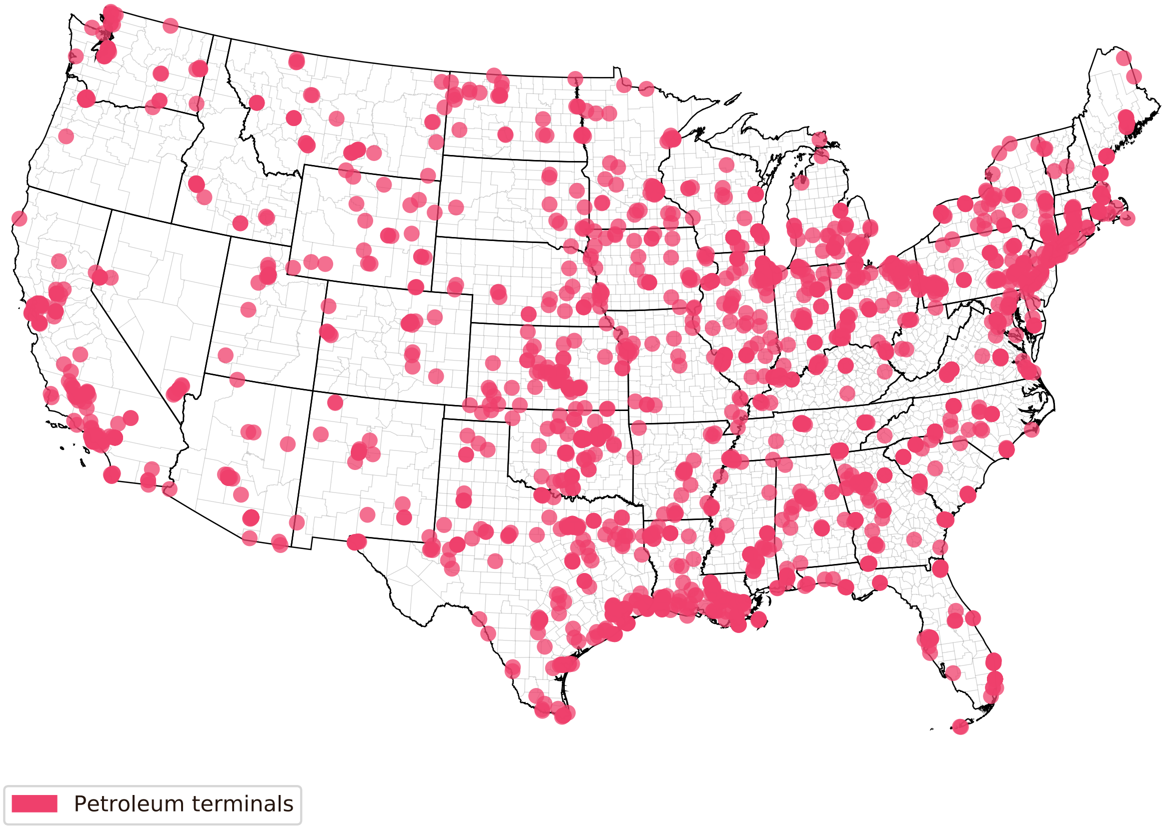

Figure S2. Map of distributed infrastructure scenario using existing petroleum terminals for product storage, related to STAR methods and all distributed scenarios.

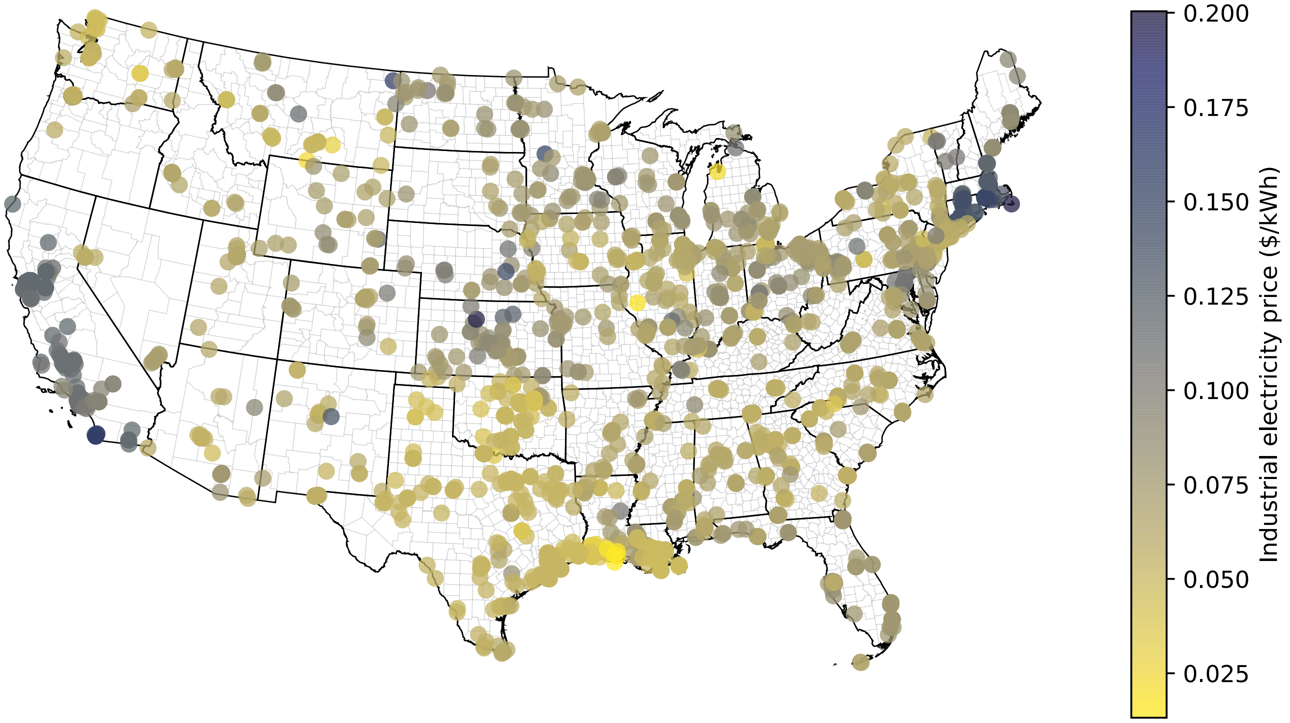

Figure S3. Industrial electricity prices at petroleum terminals, related to STAR methods.
